# The association between walking pace and hand grip strength with the risk of chronic obstructive pulmonary disease: a bidirectional Mendelian randomization study

**DOI:** 10.1186/s12890-023-02759-z

**Published:** 2023-11-20

**Authors:** Peng Qiu, Mingxian Chen, Shuaibing Lv, Juanjuan Xie, Junyu Wu

**Affiliations:** 1 Department of Rehabilitation, The First Affiliated Hospital of Wenzhou Medical University, Wenzhou, Zhejiang China; 2 School of Exercise and Health, Shanghai University of Sport, Shanghai, China; 3 School of Physical Education, Shanghai University of Sport, Shanghai, China; 4 Department of Cardiology, The First Affiliated Hospital of Wenzhou Medical University, Wenzhou, Zhejiang China

**Keywords:** Walking pace, Chronic obstructive pulmonary disease, Hand grip strength, Causal relationship, Mendelian randomization

## Abstract

**Background:**

Chronic Obstructive Pulmonary Disease (COPD) currently ranks as the third leading cause of mortality worldwide, imposing substantial burdens on societal and individual health. Amongst health research tools, walking pace (WP) and hand grip strength (HGS) are cornerstones, extensively associated with diverse health conditions. However, the intricate interplay between these factors and COPD risk remains ambiguous. This study aims to elucidate the causal association of WP, HGS, with COPD risk through a bidirectional Mendelian randomization (MR) approach.

**Methods:**

Bidirectional MR analysis was performed using Genome-wide association study (GWAS) data of European individuals for WP, HGS, and COPD. Inverse Variance Weighted (IVW) served as the primary MR analysis approach. To supplement the IVW findings, four additional MR methods [MR-Egger, weighted median, maximum likelihood, simple median] were used. To assess heterogeneity and pleiotropy, sensitivity analyses were performed. In addition, multivariate MR (MVMR) analysis was used to assess causality after adjustment for potential confounders.

**Results:**

IVW method results show a significant negative association between WP and COPD risk in both initial (genome-wide threshold, odds ratio (OR) = 0.21, 95% confidence interval (CI) 0.09–0.51, *P* = 5.06 × 10^− 4^) and secondary (locus-wide threshold, OR = 0.27, 95%CI: 0.18–0.41, *P* = 4.88 × 10^− 10^) MR analysis. The reverse MR analysis suggested that COPD also diminishes WP. Additionally, a causal risk reduction for COPD with right HGS (OR = 0.74, 95% CI: 0.58–0.94, *P* = 1.44 × 10^− 2^) was only found in secondary MR analysis. The outcomes of the four additional MR methods also suggested similar causal relationships, and sensitivity analyses endorsed their robustness. Lastly, the MVMR analysis demonstrated that the WP’s effect on reducing COPD risk persisted independently of potential confounding variables.

**Conclusion:**

A bidirectional causal relationship exists between typical WP and COPD risk. Conversely, a decrease in right HGS is unidirectionally associated with an increased risk of COPD. The study suggests that WP may serve as a predictive factor for COPD or as a simple evaluative indicator for prognosis.

**Supplementary Information:**

The online version contains supplementary material available at 10.1186/s12890-023-02759-z.

## Background

Chronic Obstructive Pulmonary Disease (COPD) represents a significant global health challenge, currently holding the position of the third most prevalent cause of mortality on a global scale [1]. Characterized by airflow obstruction, chronic inflammatory dysregulation, and emphysematous degeneration [2–4], It is typically associated with various factors such as smoking, alcohol consumption, and air pollution [5–8]. Among patients with COPD, it is estimated that 30–60% experience malnutrition [9–11], while approximately 20–40% exhibit low muscle mass [12, 13]. Alarmingly, malnutrition and muscle wasting intensify COPD prognosis and inflate healthcare costs [14, 15]. Recent research proposes a possible link between COPD and deteriorations in walking pace (WP) and hand grip strength (HGS), prevalent symptoms of sarcopenia [16, 17]. Since COPD impairs cardiopulmonary and muscular functions, it significantly undermines patients’ physical performance and overall quality of life. Consequently, early prediction of COPD becomes vital in mitigating its prevalence and attenuating its societal and individual impacts.

HGS and WP, which represent muscle strength in the upper and lower extremities, respectively, are widely accepted indicators of physical fitness. Given their utility in measuring overall physical fitness, these indicators are not only becoming increasingly important in the field of predictive epidemiology [18–22], but also show promising evidence in predicting the risk of developing diseases such as type 2 diabetes [23], cognitive decline [24], dementia [25], and cardiovascular disease [26]. Most of the current studies focus on the prognosis of COPD by WP and HGS [27–31]. While existing research has attempted to elucidate the complex relationship between these two indicators and the prevalence of COPD, a significant lacuna persists. This deficiency in our understanding emphasizes the necessity for a comprehensive exploration of their potential causality with respect to COPD risk.

Mendelian randomization (MR), which employs independent single nucleotide polymorphisms (SNPs) as instrumental variables (IVs) because of their strong correlation with exposures, facilitates the estimation of causal associations between exposures and outcomes [32]. This effectively minimizes the impact of confounding variables, eliminates reverse causality, and reduces bias. To thoroughly investigate the possible causal association of WP, HGS, with COPD, we implemented a bidirectional two-sample Mendelian randomization (MR) approach in this study [33]. By applying MR analysis, we aim to investigate whether WP and HGS provide valuable insights into the assessment of COPD risk and prognosis in COPD patients. The implications of this study could be multifaceted, potentially facilitating economical prediction of COPD, thereby providing a robust foundation for the formulation of efficacious intervention measures and therapeutic strategies. In addition, these measures may provide an uncomplicated tool for assessing the clinical prognosis of COPD patients.

## Methods

### Study design

**Fig. 1:**
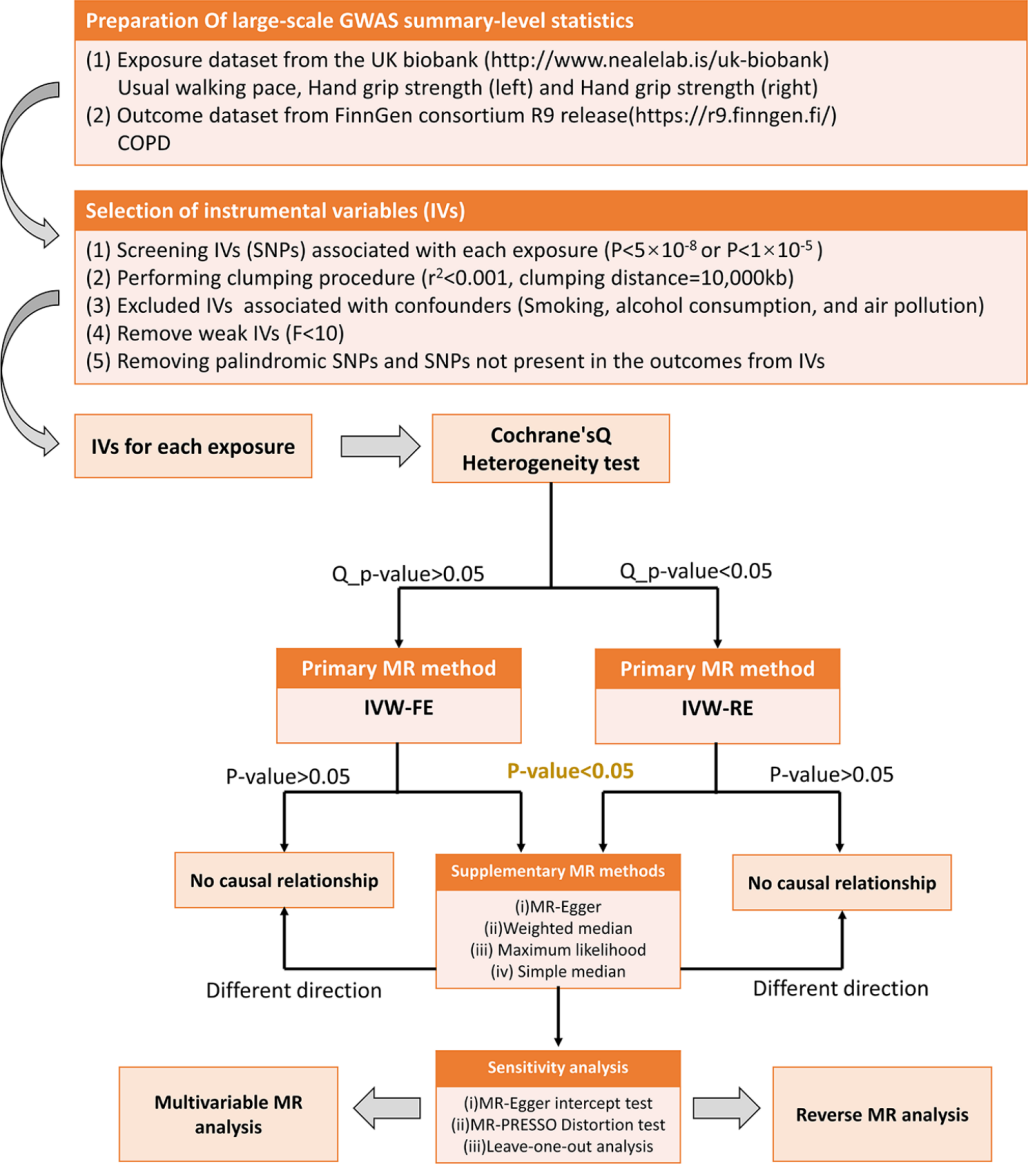
Bidirectional Mendelian Randomization study flow chart. SNP, single nucleotide polymorphism; r^2^, the linkage disequilibrium measure between SNPs; F, F-statistic; IVW-FE, inverse variance weighted (fixed effects); IVW-RE, inverse variance weighted (random effects)

We utilized MR to scrutinize the potential association of WP, HGS, with COPD. To ensure the validity of our results, we selected IVs grounded in three well-established assumptions: [1] IVs share a strong correlation with the exposure variables; [2] IVs are unrelated to any confounders; and [3] exert influence on the outcome solely via the designated exposure variables, thereby prohibiting horizontal pleiotropy. Only the IVs that meet these criteria warrant inclusion in the MR analysis [34]. Since this study is a reanalysis of previously published data, no additional ethical approval was required. The schematic representation of the overall study design can be viewed in Fig. 1.

### Data sources

The Genome-Wide Association Study (GWAS) summary statistics of WP [35], HGS (right) [36] and HGS (left) [36] were extracted from the UK biobank public database, involving 459,915, 461,089, and 461,026 European participants (GWAS ID: ukb-b-4711, ukb-b-10,215, and ukb-b-7478). For evaluating WP, participants were posed with the question, “How would you describe your usual walking pace? (Less than 3 miles per hour is a slow pace, 3–4 miles per hour is a steady average pace, and more than 4 miles per hour is a fast pace),“ included in the touchscreen questionnaire [35]. Responses were gathered from all participants apart from those who reported an inability to walk. For the measurement of HGS (right/left), the protocol was detailed in the manual available at an Assessment Centre of the UK Biobank [36]. The GWAS data for COPD [37] were procured from the publicly available database of the FinnGen Research project, published in May 2023, encompassing 18,266 cases and 311,286 controls.

### Instrumental variables selection

This MR study leveraged SNPs that displayed robust associations with each exposure as IVs. Initially, IVs were selected for the first forward MR analysis using a genome-wide significance threshold (*P* < 5 × 10^-8^) [38]. Then we performed a second forward MR analysis based on locus-wide threshold (*P* < 1 × 10^-5^) screening of IVs to increase the power of the results [39]. The fundamental rationale for using a less stringent threshold (*P* < 1 × 10^-5^) in the second MR analysis is to strike a balance between robustness (minimizing false positives) and power (enhancing the ability to detect genuine effects). By employing more genetic variants (variants with *P*-values between 5 × 10^-8^ and 1 × 10^-5^) as IVs, the statistical power of the MR analysis can be bolstered, thereby increasing the likelihood of detecting a true causal relationship between exposure and outcome [40]. We set the linkage disequilibrium correlation coefficient to r^2^ < 0.001 and adopted a clumping window size exceeding 10,000 kb to ensure IV independence. Possible confounders, such as smoking, alcohol consumption, and air pollution, could influence outcome [5–8]. Accordingly, we excluded SNPs that were associated with these confounding factors (*P* < 5 × 10^-8^) from the IVs. These confounders’ accession numbers are detailed in Table 1.

**Table 1:** Confounder source

Confounders	Source
Smoking	https://gwas.mrcieu.ac.uk/datasets/ieu-b-4877/
	https://gwas.mrcieu.ac.uk/datasets/ukb-b-223/
Alcohol consumption	https://gwas.mrcieu.ac.uk/datasets/ieu-b-73/
	https://gwas.mrcieu.ac.uk/datasets/ukb-b-5779/
Air pollution	https://gwas.mrcieu.ac.uk/datasets/ukb-b-589/
	https://gwas.mrcieu.ac.uk/datasets/ukb-b-10,817/

Subsequently, during the harmonization of the exposure and outcome statistics, we excluded palindromic and incompatible SNPs from the IVs. Also, SNPs for which corresponding exposure data could be found but for which corresponding outcome data were lacking were removed from the IVs. To minimize the impact of weak instrument bias on causal inference, we calculated the F-statistic of IVs using the formula F_exposure_ = Beta²_exposure_ / SE²_exposure_, to evaluate the strength of the IVs [41]. We eliminated those IVs with F-statistics less than 10 to prevent bias introduced by weak IVs [42].

### Statistical analyses

In our exploration of the causal relationship among WP, HGS (right/left), and COPD, we employed the inverse-variance weighted (IVW) method as the primary tool for causality analysis within the framework of MR studies [43]. The choice of using a fixed-effect IVW (IVW-FE) or a random-effect IVW (IVW-RE) hinges on the results from Cochrane’s Q heterogeneity test. If heterogeneity is detected (*P* < 0.05), we adopt the IVW-RE model, which provides a more conservative estimate. Conversely, when heterogeneity is absent, the fixed-effect IVW model is applied [44]. IVW methods based on meta-analytic principles are commonly used to perform causal analyzes in MR studies [45–47].

To strengthen the credibility and establish the directionality of our results, we utilized four additional MR methods - MR-Egger, maximum likelihood, weighted median, and simple median - for causal association assessments. The MR-Egger regression presumes that more than 50% of IVs undergo horizontal pleiotropy [48]. In contrast, the weighted median method operates under the assumption that less than 50% of IVs exhibit horizontal pleiotropy [49]. The maximum likelihood method estimates the parameters of the probability distribution by maximizing the likelihood function, typically resulting in low standard errors [50]. In scenarios where a significant fraction of genetic variants is deemed invalid as instrumental variables, the method can deliver consistent causal effects [51]. We acknowledged only those exposure-outcome pairs with uniform directional implications across all MR methods as possessing a causal association.

A succession of sensitivity analyses was conducted to evaluate the reliability of MR results. Initially, we applied the MR-Egger intercept for the detection of horizontal pleiotropy [48, 52]. Moreover, we employed the MR-PRESSO distortion test, an integral component of the MR-PRESSO framework, to ascertain whether MR estimates remained consistent post the elimination of potential pleiotropic outliers [52]. We then performed a leave-one-out analysis to evaluate whether any single SNP significantly influenced or biased the MR estimate. In this analysis, each SNP is sequentially excluded from the IVs, and the causal effect is re-estimated without that particular SNP. If the MR estimate changes significantly upon the exclusion of a particular SNP, it suggests that this SNP might have an influence on the overall result. Conversely, if the MR estimates remain consistent across all iterations, it indicates that the findings are not biased by any individual SNP.

We set the threshold for statistical significance at *P* < 0.05. Odds ratios (OR) with 95% confidence intervals (95% CI) were then used to present the results of causal associations. These analyses were executed utilizing the “TwoSampleMR” (version 0.5.6) [53] and “MRPRESSO” (version 1.0) [54] packages in R software (version 4.2.3).

### Reverse mendelian randomization analysis

A reverse MR analysis was undertaken to examine the potential causal influence of COPD on WP and HGS (right/left). Due to the limited number of SNPs (*P* < 5 × 10^− 8^) within the COPD GWAS summary^3^ data, we selected SNPs (*P* < 1 × 10^− 5^) as IVs. The protocols described in the “Instrumental variables selection” and “Statistical analyses” sections were followed for subsequent procedures.

### Multivariable mendelian randomization analysis

MVMR is an extension of the standard MR approach that allows for the simultaneous evaluation of multiple exposures on an outcome. This method is particularly useful when there’s a need to adjust for potential confounders or when examining the combined effects of multiple exposures [45]. For pairs of exposure outcomes that were found to be significant in the univariate MR analysis at IV (*P* < 5 × 10^− 8^), we performed a multivariate MR (MVMR) approach [45]. This study factored in three potential confounders: smoking, alcohol consumption, and air pollution (IEU GWAS ID: ieu-b-4877, ukb-b-5779, and ukb-b-589).

Following the consolidation of GWAS summary datasets for the exposure and these confounders, we ensured each IV sustained a robust correlation (*P* < 5 × 10^− 8^) with at least one exposure or confounder. SNPs were pruned within a 10,000 kb window using a threshold of r^2^ < 0.001 to reduce the effects of LD. The IVW method was applied to evaluate causal effects after removal of palindromic SNPs and SNPs not present in the outcome data, taking these confounders into account.

## Results

### MR analysis results based on IVs of genome-wide significance screen

The MR results detailed in this section were derived from IVs filtered based on the genome-wide significance threshold (*P* < 5 × 10^− 8^). Following the exclusion of 11, 7, and 8 SNPs associated with confounders from the IVs of WP, right HGS, and left HGS, along with the removal of SNPs absent in the outcome data and palindromic SNPs, we proceeded to investigate the causal impacts of WP, right HGS, and left HGS on COPD, employing 42, 149, and 129 IVs correspondingly. Comprehensive information on confounder SNPs is provided in Additional File 1: Table S1, and specifics of MR analysis IVs are provided in Additional File 1: Table S2. The F statistic of each IV ranges from 29.78 to 231.74.

**Fig. 2:**
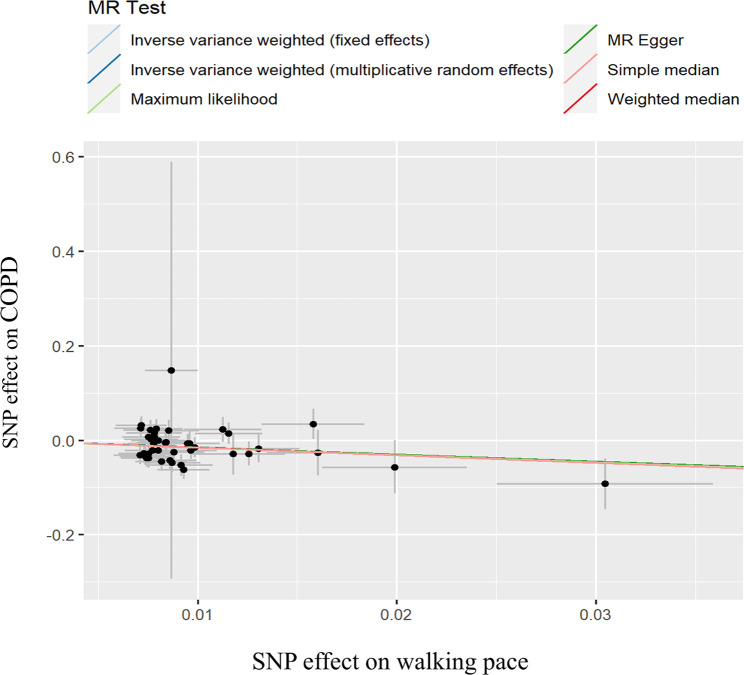
Scatter plot of genetic associations of walking pace on COPD

Initially revealed heterogeneity in both WP and left HGS through Cochrane’s Q test (*P* < 0.05; Table 2), which necessitated the use of the IVW-RE model to furnish a more conservative estimate. The MR findings suggest a causal relationship between WP and COPD, though a similar causal association between HGS (right/left) and COPD was not evident. Specifically, the MR results denoted an association between WP and a decreased risk of COPD (OR_IVW−RE_= 0.21, 95%CI: 0.09–0.51, *P* = 5.06 × 10^− 4^). These findings are consistent across other MR methods, corroborating the IVW results, though MR Egger was not significant, which did not affect the overall results (Table 3). In Fig. 2, scatter plots offer an intuitive representation of the causal relationship between the genetic association of exposure (WP) on the x-axis and the outcome (risk of COPD) on the y-axis. The best fit for each point in the scatter plot, representing an SNP, is denoted by the diagonal, commonly referred to as the MR regression line. The slope of this line indicates the estimated causal effect; a downward slope suggests that as the genetic susceptibility to WP increases, the risk of COPD decreases.

**Table 2:** Results of sensitivity analyses (genome-wide significance threshold)

Exposure	Outcome	Cochran’s Q test				Pleiotropy				
		MR-Egger		IVW		MR Egger			MR-PRESSO Distortion Test	
		Q	Q-pval	Q	Q-pval	intercept	SE	*P*-value	n Outliers	*P*-value
WP	COPD	58.509	0.030	58.512	0.037	-0.001	0.016	0.968	NA	NA
HGS (right)	COPD	173.347	0.068	174.247	0.069	0.006	0.007	0.384	NA	NA
HGS (left)	COPD	167.864	0.009	168.106	0.010	-0.004	0.008	0.669	1	0.557

WP, walking pace; HGS, Hand grip strength; COPD, chronic obstructive pulmonary disease; IVW, inverse variance weighted; SE, standard error

**Table 3:** MR results of walking pace and hand grip strength (right/left) on COPD (genome-wide significance threshold)

Exposure	Outcome	n SNP	Method	OR (95% CI)	*P*-value
WP	COPD	42	IVW-FE	0.21(0.10,0.44)	3.26E-05
			IVW-RE	0.21(0.09,0.51)	5.06E-04
			MR Egger	0.23(0.01,8.28)	4.23E-01
			Weighted median	0.21(0.06,0.67)	8.82E-03
			Maximum likelihood	0.21(0.10,0.45)	6.12E-05
			Simple median	0.20(0.06,0.63)	5.94E-03
HGS (right)	COPD	149	IVW-FE	0.87(0.65,1.17)	3.51E-01
			IVW-RE	0.87(0.63,1.20)	3.90E-01
			MR Egger	0.52(0.15,1.74)	2.87E-01
			Weighted median	0.84(0.52,1.34)	4.55E-01
			Maximum likelihood	0.87(0.64,1.17)	3.61E-01
			Simple median	1.00(0.64,1.56)	1.00E + 00
HGS (left)	COPD	129	IVW-FE	0.84(0.61,1.16)	2.98E-01
			IVW-RE	0.84(0.58,1.22)	3.64E-01
			MR Egger	1.14(0.28,4.64)	8.60E-01
			Weighted median	0.81(0.50,1.33)	4.04E-01
			Maximum likelihood	0.84(0.61,1.17)	3.06E-01
			Simple median	0.85(0.52,1.38)	5.09E-01

WP, walking pace; HGS, Hand grip strength; COPD, chronic obstructive pulmonary disease; IVW-FE, inverse variance weighted (fixed effects); IVW-RE, inverse variance weighted (random effects); OR, Odds ratios; 95% CI, 95% confidence intervals

In the sensitivity analysis, the MR-Egger intercept test results showed no apparent influence of horizontal pleiotropy in the WP analysis (*P* = 0.968; Table 2). Moreover, the MR-PRESSO detected one outlier (rs2587505) in the left HGS analysis, and Distortion Test show the association persisted even after excluding this SNP (*P* = 0.557; Table 2). Lastly, the robustness of the MR findings was verified by the leave-one-out sensitivity analysis, confirming no single SNP significantly skewed the results upon removal (Additional file 2: Figure S1).

### MR analysis results based on IVs of the locus -wide significance

To increase the robustness of our findings, a second MR analysis was performed with IV (*P* < 1 × 10^− 5^). Following the exclusion of 19, 9, and 10 SNPs associated with confounders from the IVs of WP, right HGS, and left HGS, along with the removal of SNPs absent in the outcome data and palindromic SNPs, we proceeded to investigate the causal impacts of WP, right HGS, and left HGS on COPD, employing 222, 347, and 348 IVs correspondingly. Detailed information on confounder SNPs is provided in Additional File 1: Table S3, and specifics of MR analysis IVs are provided in Additional File 1: Table S4. The F statistic of each IV ranges from 19.53 to 231.74.

**Fig. 3:**
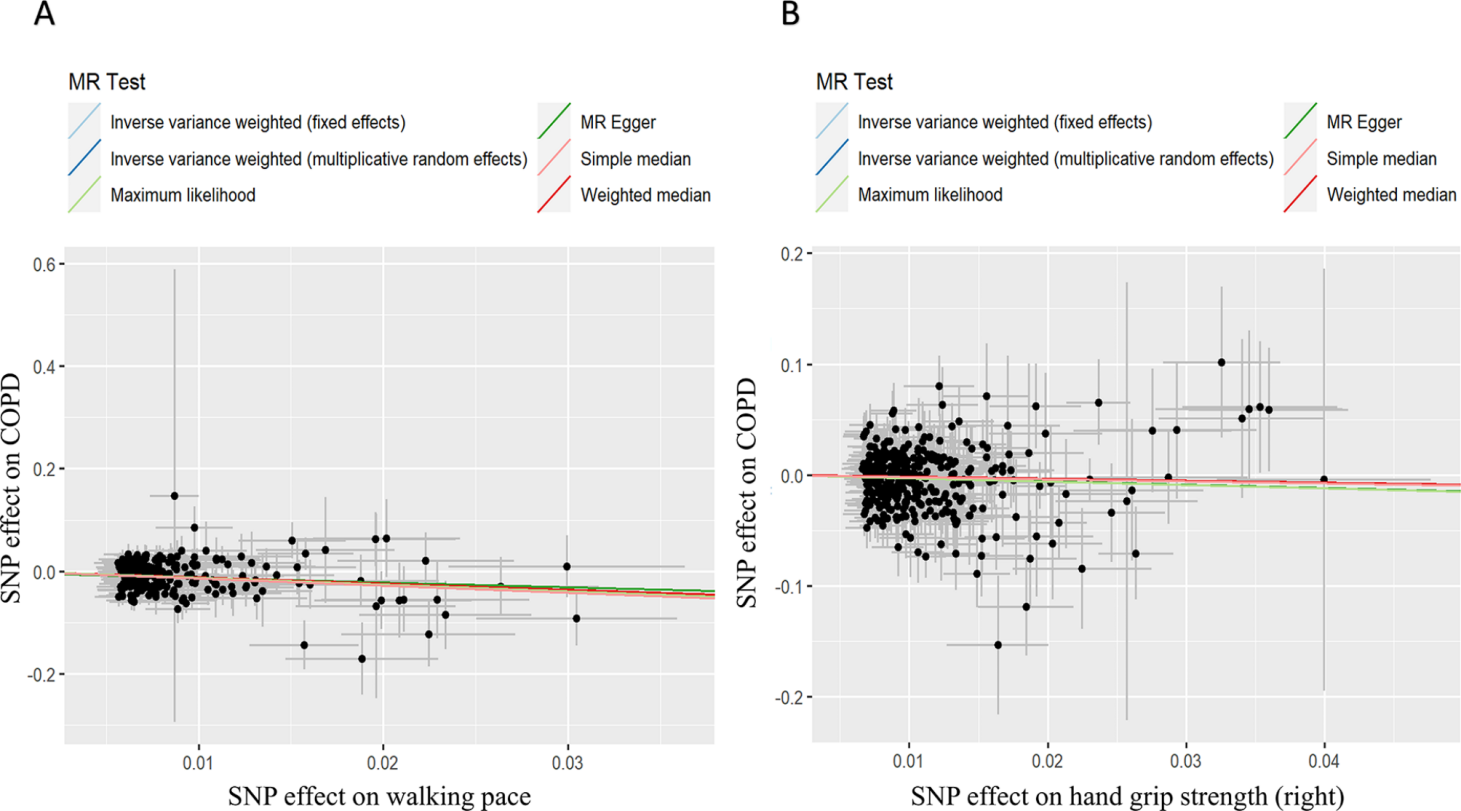
Scatter plot of genetic associations between exposure and outcome. (**A**) walking pace and COPD. (**B**) hand grip strength (right) and COPD

Initially, heterogeneity in WP and HGS (right/left) was initially detected, substantiated by Cochrane’s Q test results (*P* < 0.05; Table 4). Subsequently, the IVW-RE model was adopted to offer a more conservative estimate. The MR results in this section were consistent with the previous section, with a causal relationship between WP and COPD risk reduction (OR_IVW−RE_ = 0.27, 95%CI: 0.18–0.41, *P* = 4.88 × 10^− 10^). Importantly, right HGS was also found to causally reduce the risk of COPD (OR_IVW−RE_ = 0.74, 95% CI: 0.58–0.94, *P* = 1.44 × 10^− 2^; Table 5; Fig. 3).

**Table 4:** Results of sensitivity analyses (locus-wide significance threshold)

Exposure	Outcome	Cochran’s Q test				Pleiotropy				
		MR-Egger		IVW		MR Egger			MR-PRESSO Distortion Test	
		Q	Q-pval	Q	Q-pval	intercept	SE	*P*-value	n Outliers	*P*-value
WP	COPD	259.524	0.035	259.872	0.037	-0.003	0.006	0.588	NA	NA
HGS (right)	COPD	391.007	0.044	391.007	0.048	-0.001	0.004	0.994	NA	NA
HGS (left)	COPD	402.763	0.019	403.808	0.019	0.004	0.005	0.344	2	0.029

WP, walking pace; HGS, Hand grip strength; COPD, chronic obstructive pulmonary disease; IVW, inverse variance weighted; SE, standard error

**Table 5:** MR results of walking pace and hand grip strength (right/left) on COPD (locus-wide significance threshold)

Exposure	Outcome	n SNP	Method	OR (95% CI)	*P*-value
WP	COPD	222	IVW-FE	0.27(0.18,0.39)	1.50E-11
			IVW-RE	0.27(0.18,0.41)	4.88E-10
			MR Egger	0.40(0.09,1.76)	2.27E-01
			Weighted median	0.30(0.17,0.56)	1.17E-04
			Maximum likelihood	0.27(0.18,0.40)	5.05E-11
			Simple median	0.25(0.14,0.44)	1.73E-06
HGS (right)	COPD	347	IVW-FE	0.74(0.59,0.93)	9.26E-03
			IVW-RE	0.74(0.58,0.94)	1.44E-02
			MR Egger	0.74(0.33,1.68)	4.71E-01
			Weighted median	0.84(0.58,1.21)	3.44E-01
			Maximum likelihood	0.74(0.59,0.93)	1.10E-02
			Simple median	0.83(0.59,1.16)	2.77E-01
HGS (left)	COPD	348	IVW-FE	1.00(0.79,1.26)	9.74E-01
			IVW-RE	1.00(0.78,1.28)	9.76E-01
			MR Egger	0.66(0.27,1.60)	3.60E-01
			Weighted median	0.99(0.70,1.42)	9.77E-01
			Maximum likelihood	1.00(0.79,1.26)	9.75E-01
			Simple median	1.06(0.74,1.52)	7.51E-01

WP, walking pace; HGS, Hand grip strength; COPD, chronic obstructive pulmonary disease; IVW-FE, inverse variance weighted (fixed effects); IVW-RE, inverse variance weighted (random effects); OR, Odds ratios; 95% CI, 95% confidence intervals

Within the sensitivity analysis, horizontal pleiotropy in the MR-Egger intercept test had no significant impact on the MR analysis of WP (*P* = 0.588) and right HGS (*P* = 0.994; Table 4). Additionally, MR-PRESSO identified two outliers (rs2594205 and rs6587138) within the left HGS analysis (Table 4). Lastly, the robustness of the MR findings was verified by the leave-one-out sensitivity analysis, confirming no single SNP significantly skewed the results upon removal (Additional file 2: Figure S2).

### Results of reverse mendelian randomization analysis

In order to assess whether COPD exerts a causal effect on WP and HGS (right/left), a reverse MR analysis was conducted. Initially, 4 SNPs excluded from COPD IVs associated with confounders (smoking and alcohol consumption). Subsequently, the causal impacts of COPD on WP and HGS (right/left) were evaluated using 37 IVs (*P* < 1 × 10^− 5^), following the exclusion of absent SNPs in the outcome and palindromic SNPs. Comprehensive information on confounder SNPs can be found in Additional File 1: Table S5, and specifics of reverse MR analysis IVs are provided in Additional File 1: Table S6. The F statistic of each IV ranges from 19.65 to 120.42.

**Table 6:** MR results of COPD on walking pace and hand grip strength (right/left)

Exposure	Outcome	n SNP	Method	OR (95% CI)	*P*-value
COPD	WP	37	IVW-FE	0.99(0.99,1.00)	1.31E-03
			IVW-RE	0.99(0.99,1.00)	1.06E-02
			MR Egger	0.99 (0.99,1.01)	4.43E-01
			Weighted median	0.99(0.99,1.00)	8.20E-02
			Maximum likelihood	0.99(0.99,1.00)	1.72E-03
			Simple median	0.99(0.99,1.00)	7.82E-02
COPD	HGS (right)	37	IVW-FE	1.00(0.99,1.00)	3.00E-01
			IVW-RE	1.00(0.99,1.00)	4.21E-01
			MR Egger	0.99(0.98,1.01)	3.13E-01
			Weighted median	0.99(0.99,1.00)	1.44E-01
			Maximum likelihood	1.00(0.99,1.00)	3.06E-01
			Simple median	1.00(0.99,1.00)	4.45E-01
COPD	HGS (left)	37	IVW-FE	1.00(0.99,1.00)	3.28E-01
			IVW-RE	1.00(0.99,1.00)	4.88E-01
			MR Egger	0.99(0.98,1.00)	1.26E-01
			Weighted median	1.00(0.99,1.00)	2.63E-01
			Maximum likelihood	1.00(0.99,1.00)	3.21E-01
			Simple median	1.00(0.99,1.00)	3.14E-01

WP, walking pace; HGS, Hand grip strength; COPD, chronic obstructive pulmonary disease; IVW-FE, inverse variance weighted (fixed effects); IVW-RE, inverse variance weighted (random effects); OR, Odds ratios; 95% CI, 95% confidence intervals

**Fig. 4:**
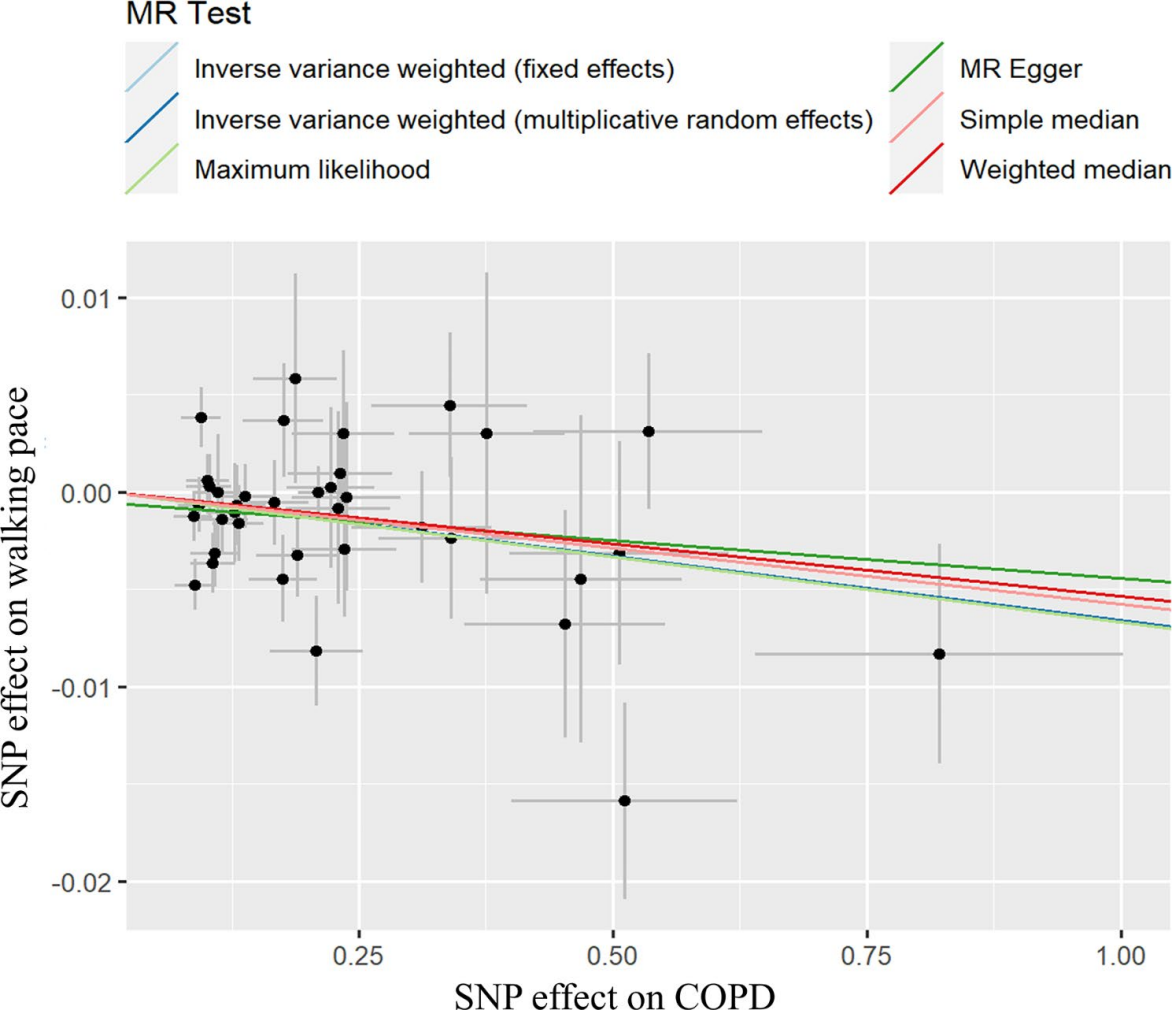
Scatter plot of genetic associations of COPD on walking pace from reverse MR analysis

Heterogeneity affected the reverse MR analysis according to Cochran’s Q test (*P* < 0.05; Table 7), Subsequently, the IVW-RE model was adopted to offer a more conservative estimate. The outcomes from the MR methods suggest a causal influence of COPD on a decrease in WP (OR_IVW−RE_ = 0.99, 95%CI: 0.99–1.00, *P* = 1.06 × 10^− 2^; Table 6; Fig. 4). Moreover, the MR-Egger intercept test showed that horizontal pleiotropy did not have a significant effect in the MR analysis of COPD on WP (*P* = 0.532; Table 7). Furthermore, MR-PRESSO identified one (rs2199036) and two (rs2199036 and rs8040868) outliers in the analysis for right and left HGS, respectively, with the association persisting post exclusion of these SNPs (*P*_right HGS_ = 0.773; *P*_left HGS_ = 0.430; Table 7). Lastly, the robustness of the reverse MR results was validated by the leave-one-out sensitivity analysis. (Additional file 2: Figure S3).

**Table 7:** Results of sensitivity analyses in reverse MR analysis

Exposure	Outcome	Cochran’s Q test				Pleiotropy				
		MR-Egger		IVW		MR Egger			MR-PRESSO Distortion Test	
		Q	Q-pval	Q	Q-pval	intercept	SE	*P*-value	n Outliers	*P*-value
COPD	WP	56.216	0.013	56.855	0.015	-0.001	0.001	0.532	NA	NA
COPD	HGS (right)	58.711	0.007	59.560	0.008	0.001	0.001	0.481	1	0.773
COPD	HGS (left)	68.004	0.001	71.850	0.001	0.002	0.001	0.168	2	0.430

WP, walking pace; HGS, Hand grip strength; COPD, chronic obstructive pulmonary disease; IVW, inverse variance weighted; SE, standard error

### Results of multivariable mendelian randomization analysis

**Fig. 5:**
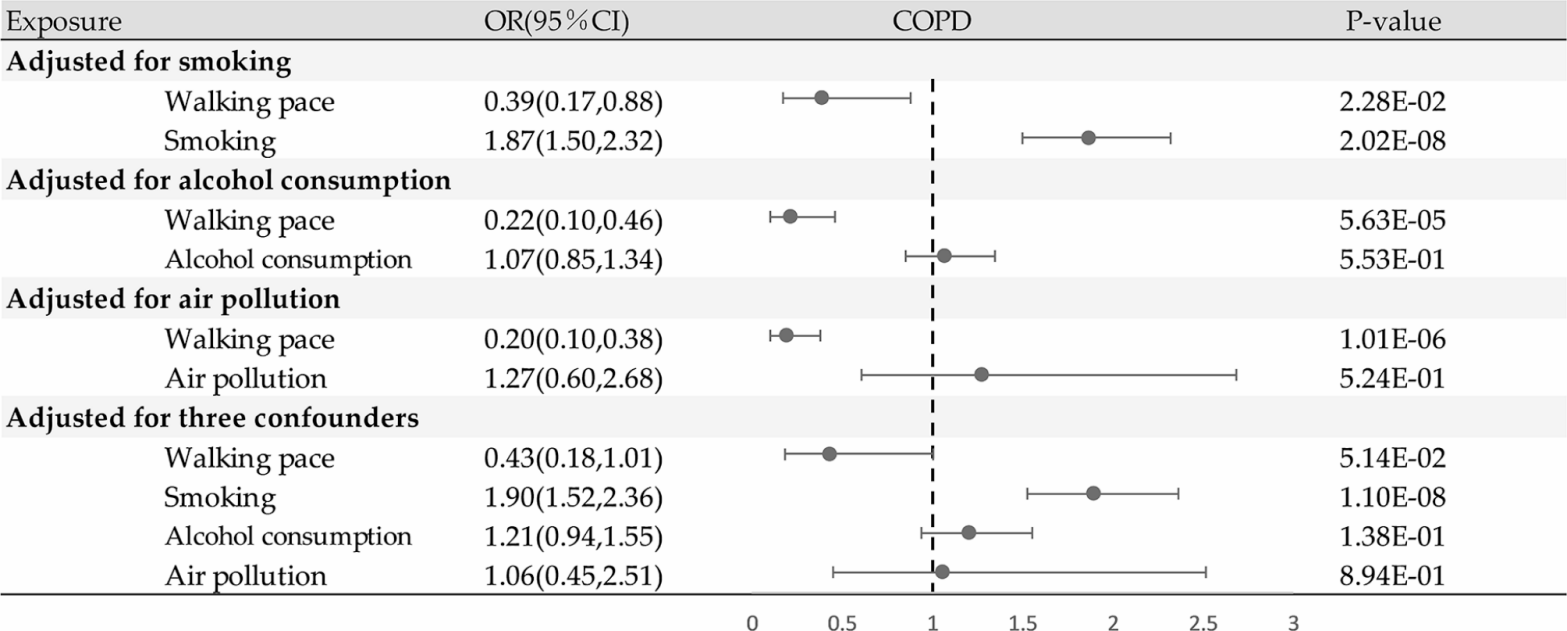
MVMR analysis for assessing the causal effect of walking pace on COPD

A supplementary MVMR analysis was performed to further evaluate the causal estimations of WP on COPD. This analysis considered three confounders: smoking, alcohol consumption, and air pollution. After adjusting for smoking (OR = 0.39, 95%CI: 0.17–0.88, *P* = 2.28 × 10^− 2^), alcohol consumption (OR = 0.22, 95% CI: 0.10–0.46, *P* = 5.63 × 10^− 5^), and air pollution (OR = 0.20, 95% CI: 0.10–0.38, *P* = 1.01 × 10^− 6^), WP persistently exhibited a causal association with a decreased risk of COPD (Fig. 5). This effect was more substantial compared to the causal relationship identified through the univariate MR analysis. After adjusting for the three confounders, WP consistently showed a causal relationship with a reduced risk of COPD, although the association was not statistically significant (OR = 0.43, 95%CI: 0.18–1.01, *P* = 5.14 × 10^− 2^; Fig. 5).

## Discussion

Through various MR investigations, we have identified a bidirectional causal relationship between WP and COPD risk, whereas HGS showed a unidirectional causal link from HGS (right) to COPD only. The MVMR shows that our research results remain stable even after adjustment for confounders such as smoking, alcohol consumption and air pollution. Specifically, at the genome-wide significance threshold (*P* < 5 × 10^− 8^), only WP was observed to be causally related to COPD, suggesting that slower WP may be associated with higher COPD risk. Additionally, when we lowered the threshold to *P* < 1 × 10^− 5^, there is a causal relationship between WP and the risk of COPD. Moreover, we identified a correlation between increased COPD risk and decreased HGS (right), with no such link present for HGS (left). These findings reinforce the potential of using WP as a screening indicator for COPD risk, and further affirm its prognostic value in the context of COPD. Nevertheless, caution is warranted when considering the use of HGS as a predictive marker for COPD risk.

From the direction of WP and HGS to COPD, this study found a potential causal relationship between WP, HGS (right) and COPD. Currently, WP has been elevated to the status of the ‘sixth vital sign’ [55], following heart rate, body temperature, respiration, pain, and blood pressure. Existing literature on this interrelation between WP and COPD remains relatively scant, with the majority of research focusing on evaluating the WP of diagnosed COPD patients to predict various outcomes including physical activity level [27], movement ability [28, 29], nutritional status, and sarcopenia. Although these studies underscore the prognostic value of WP for individuals with COPD, the inherent design of these investigations, which predominantly involve participants already diagnosed with COPD, the available evidence does not provide a clear understanding of the causal relationship between WP and COPD risk. Specifically, it remains uncertain whether COPD is a consequence of reduced WP or if the reverse is true. Our study employs the bidirectional two-sample MR approach, which effectively accounts for the underlying causal relationship.

Our MVMR consistently emphasized the protective effect of WP against COPD risk, after independently considering confounding factors detrimental to lung health, such as smoking, alcohol consumption, and air pollution [5–8]. Furthermore, even after collectively adjusting for these confounders, WP maintained a trend of protection against COPD, although it wasn’t statistically significant. This consistent protective effect of WP, independent of the confounders, accentuates its significance in strategies aimed at reducing COPD risk. While current literature seldom addresses the impact of reduced WP on COPD risk, existing research still advocates for the use of WP as a simple and effective indicator for distinguishing the risk factor associated with COPD [56]. Given that WP encapsulates the overall status of an individual and is connected to numerous health outcomes [57], combined with the multifactorial pathogenesis of COPD [58, 59], the precise mechanistic link between the two remains unclear. Here are a few potential mechanisms for the increased risk of COPD due to slower WP. Firstly, WP to some extent reflects an individual’s level of physical activity. Typically, higher levels of physical activity correlate with faster WP. However, long-term sedentary behavior could result in inadequate exercise for both lung and muscle functions, leading to their deterioration and subsequently increasing the risk of COPD [60–62]. Secondly, WP can serve as an indicator of an individual’s general physical functional capability [57], particularly when the immune system and lung function are compromised, increasing the risk of COPD [63]. Furthermore, slow WP is generally associated with aging and the presence of other comorbidities such as cardiovascular disease or musculoskeletal issues [58]. Both aging and the existence of other chronic diseases could be risk factors for the development of COPD. These potential mechanisms underscore the complexity of the relationship between WP and COPD risk, indicating the necessity for further research to better understand the underlying mechanisms.

When the threshold was lowered to (*P* < 1 × 10^− 5^) in the direction from WP and HGS to COPD risk, a causal relationship was observed between HGS (right) and COPD risk. Intriguingly, changes in COPD risk appeared to be unrelated to HGS (left). Several factors might contribute to this phenomenon. First, previous research has shown that most individuals exhibit a preference for their right hand [64]. This dominant hand is frequently engaged to maintain basic functional activities in daily life, such as brushing teeth, eating, and writing, among others. The habitual use of the dominant hand helps maintain its muscular strength. Thus, a noticeable decline in the muscle strength of the dominant hand may indicate a greater risk of disease. Additionally, it has been demonstrated that central neural control may differ between the dominant and non-dominant hands [65]. These neural discrepancies could potentially influence the strength and functionality of each hand differently, which might explain the observed differences in the potential causal relationships between HGS (right/left) and COPD risk. As a result, any noticeable decline in the muscle strength of the dominant hand might be more indicative of overall health deterioration, including increased risk of diseases like COPD. Nevertheless, at the time of our analysis, no GWAS database on the dominant hand was found, so we did not directly adjust for handedness in the MR model. given that the set threshold is not the most stringent, and considering the complex interrelated mechanisms between HGS and COPD risk, these results should be interpreted with caution. Future research is required to explore these mechanisms and validate these preliminary findings further.

From the direction of COPD to WP and HGS, our study identified potential causal associations linking COPD risk with WP. Our findings align with previous research which affirmed the prognostic value of WP, particularly evident in its capacity to identify high-risk COPD patients and its role in evaluating the physical capabilities of individuals with COPD [56, 66, 67]. The 4-meter WP test has emerged as a convenient tool for assessing the physical function of patients with clinical COPD [68]. Moreover, earlier studies indicate that over 20% of COPD patients were diagnosed with malnutrition, which was linked with a significant decline in their WP [66]. Concurrently, another study found that the average WP for ambulatory COPD patients was merely 0.91 m/s. Given that the minimum speed to safely cross a pedestrian crosswalk is 1.2 m/s, it was observed that only around 10% of COPD patients were able to meet this threshold [67]. Another significant consideration stems from the defining characteristics of COPD, which include progressive airflow limitation and pulmonary inflammation [69]. The resulting impairment in lung function could lead to a decline in physical endurance, consequently leading to a slower gait speed. Simultaneously, recent research suggests that the decrease in WP among COPD patients seems to be attributed not only to peripheral changes related to sarcopenia, but more likely due to the loss of neural autonomic function [70]. In this context, slower gait speed would be indicative of a symptom rather than a causative factor of COPD. Finally, given that COPD is a systemic rather than a localized inflammation, it is plausible that COPD may lead to a decrease in WP through complications or by affecting the functionality of other organs. This chain of logical connections provides a comprehensive understanding of our findings and their implications, further emphasizing the complexities inherent in the exploration of these associations.

This research has multiple strengths. Predominantly, it introduces, to our knowledge, the first instance of the bidirectional two-sample MR approach to unearth potential causal connections among WP, HGS, and COPD risk. Our methodology importantly merges two unique exposure measures - WP and HGS, and undertakes a bidirectional investigation. This thorough methodology paves the way for an intricate examination of the relationships between these factors and COPD risk. In the second place, the two-sample MR analysis utilizes independently summarized data from substantial GWAS. The considerable sample size of these studies fortifies the reliability of our conclusions. Thirdly, we put in place strict criteria for the selection of instrumental variables, deeming a causal relationship credible only if confirmed by multiple MR methods. To examine the reliability of our conclusions, we utilized a suite of MR techniques and conducted thorough sensitivity analyses. Finally, our pinpointing of WP as a potential preliminary screening tool for COPD risk could greatly economize community screenings. Additionally, the evaluation of WP allows individuals to intuitively discern their functional status and level of physical activity. This provides a vital window for exercise intervention or the promotion of healthier lifestyles.

In addition to the strengths identified in this study, there are certain limitations that should be acknowledged. First, our findings may not be generalizable to all ethnic groups because the analysis used GWAS summary data from European populations. Second, stratified analysis based on common factors (age, sex, etc.) was not possible given the limitations of the summarized GWAS data. Thirdly, there is no guarantee that there are no pleiotropic effects in the study. Therefore, we performed sensitivity analyses to validate the reliability of our finding. Furthermore, in the reverse MR analysis, we used a relatively relaxed threshold (*P* < 1 × 10^− 5^) for IV selection due to the small number of IVs meeting the strict threshold (*P* < 5 × 10^− 8^), which may impose limitations on the stability of the results.

## Conclusion

Our study stands as the first to employ a bidirectional two-sample MR in revealing the association of WP, HGS, with COPD risk, and our findings demonstrate a reciprocal causal relationship between WP and COPD risk. Concurrently, we also uncovered a potential causal relationship between the decrease in HGS (right) and the increase in COPD risk. Hence, our research provides preliminary evidences for the utilize of WP as indicator for predicting COPD risk, yet caution is still required when implementing the HGS index. Predicting COPD risk via WP may help reduce diagnostic costs during initial community screening and serve as the first step in promoting physical health awareness by enabling individuals to understand their own functional abilities. Further research is necessary to elucidate the biological mechanisms underpinning the association between WP and COPD, with the aim of determining the most efficacious clinical intervention strategies.

### Electronic supplementary material

Below is the link to the electronic supplementary material.

Supplementary Material 1

Supplementary Material 2

## Data Availability

The GWAS data of walking pace was retrieved from IEU-Open GWAS project (https://gwas.mrcieu.ac.uk/datasets/ukb-b-4711/) online platform. The GWAS data of Hand grip strength (right/left) was retrieved from IEU-Open GWAS project (https://gwas.mrcieu.ac.uk/datasets/ukb-b-10215/ and https://gwas.mrcieu.ac.uk/datasets/ukb-b-7478/) online platform. The GWAS data of Chronic obstructive pulmonary disease were retrieved from IEU-Open GWAS project (https://gwas.mrcieu.ac.uk/datasets/finn-b-J10_COPD/).

## Abbreviations

MR: Mendelian randomization
WP: Walking pace
HGS: Hand grip strength
COPD: Chronic obstructive pulmonary disease
IVW-FE: Fixed-effect inverse variance weighted model
IVW-MRE: Multiplicative random-effect inverse variance weighted model
GWAS: Genome-wide association study
