# Supplementary material for: The association between walking pace and hand grip strength with the risk of chronic obstructive pulmonary disease: a bidirectional Mendelian randomization study
Source: BMC Pulm Med. 2023 Nov 20;23:450. doi: 10.1186/s12890-023-02759-z (PMC10658936; doi:10.1186/s12890-023-02759-z)
Supplement: Supplementary file 2 — Supplementary Material 2 [file 12890_2023_2759_MOESM2_ESM.docx]

Supplementary Figures

The Association between Walking Pace and Hand Grip Strength with the Risk of Chronic Obstructive Pulmonary Disease: A Bidirectional Mendelian Randomization Study

**Figure S1.** Leave-one-out sensitivity analysis under genome-wide significance threshold instrumental variables: (A) walking pace on COPD; (B) hand grip strength (right) on COPD; (C) hand grip strength (left) on COPD.


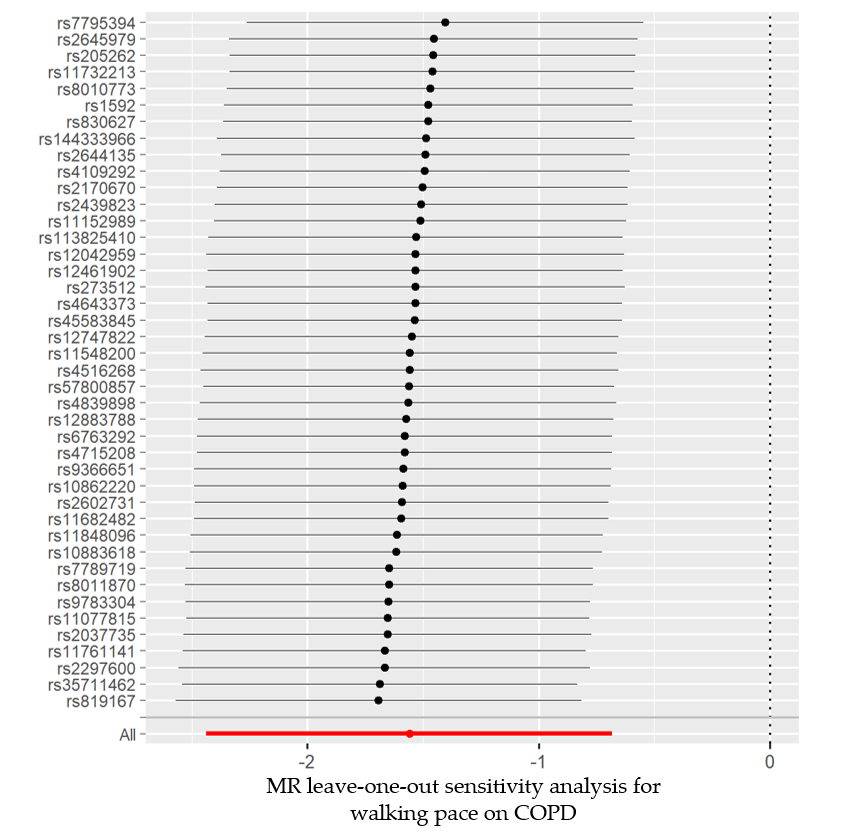


(A)


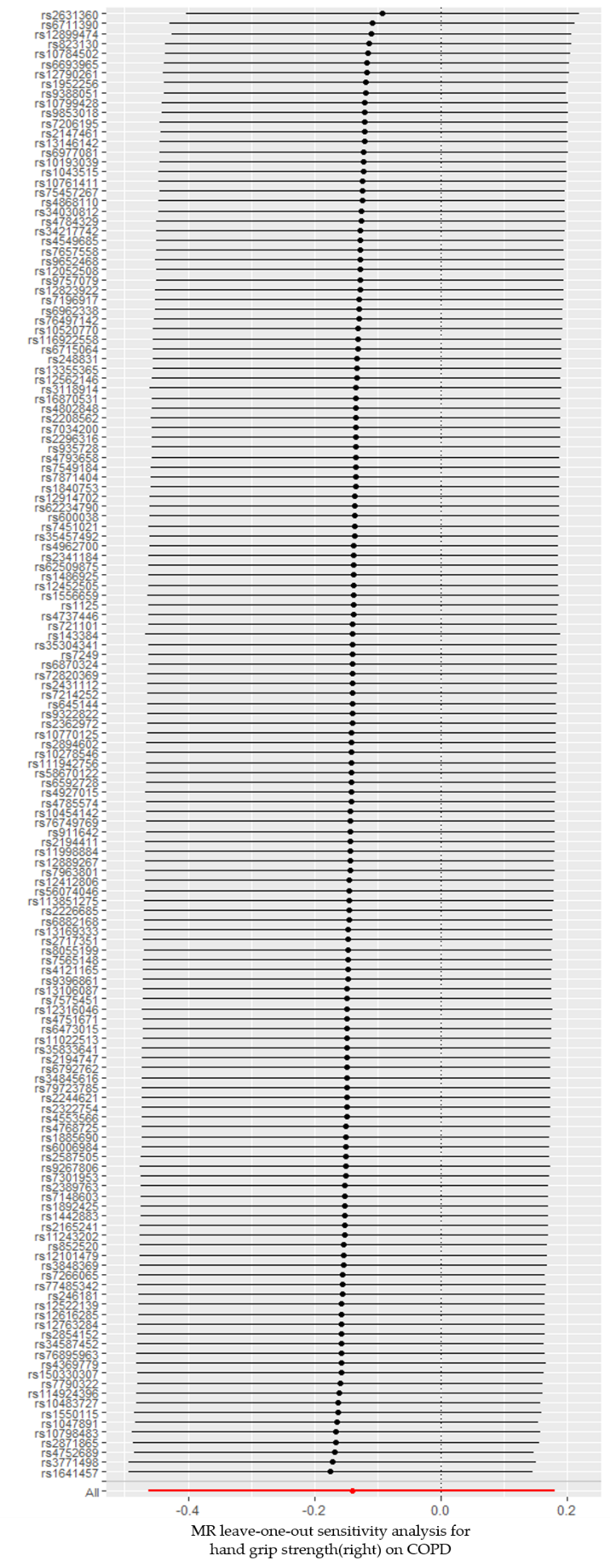


(B)


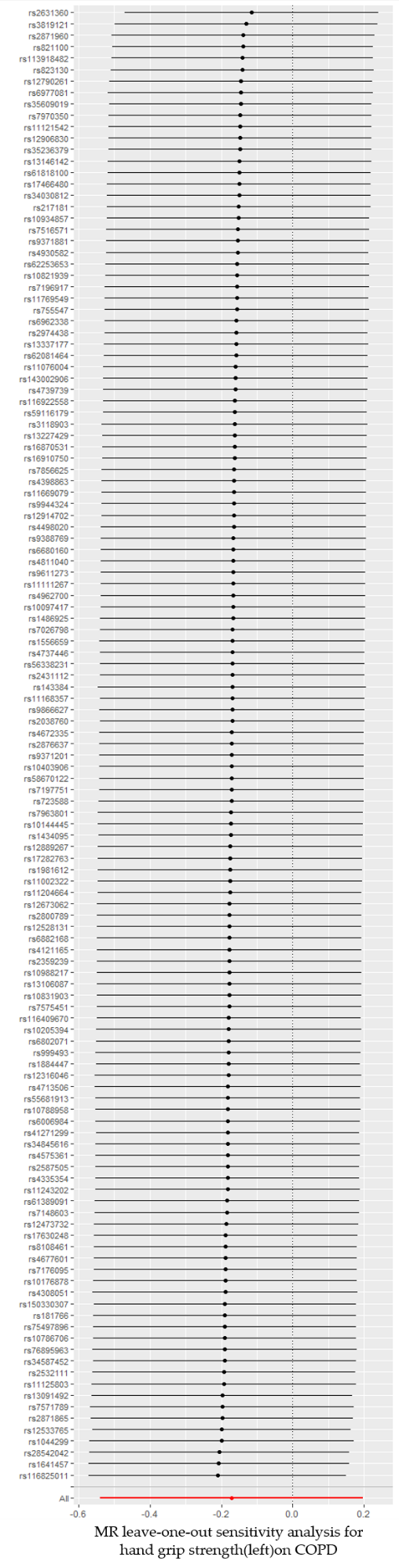


(C)

**Figure S2.** Leave-one-out sensitivity analysis under locus-wide significance threshold instrumental variables: (A) walking pace on COPD; (B) hand grip strength (right) on COPD; (C) hand grip strength (left) on COPD


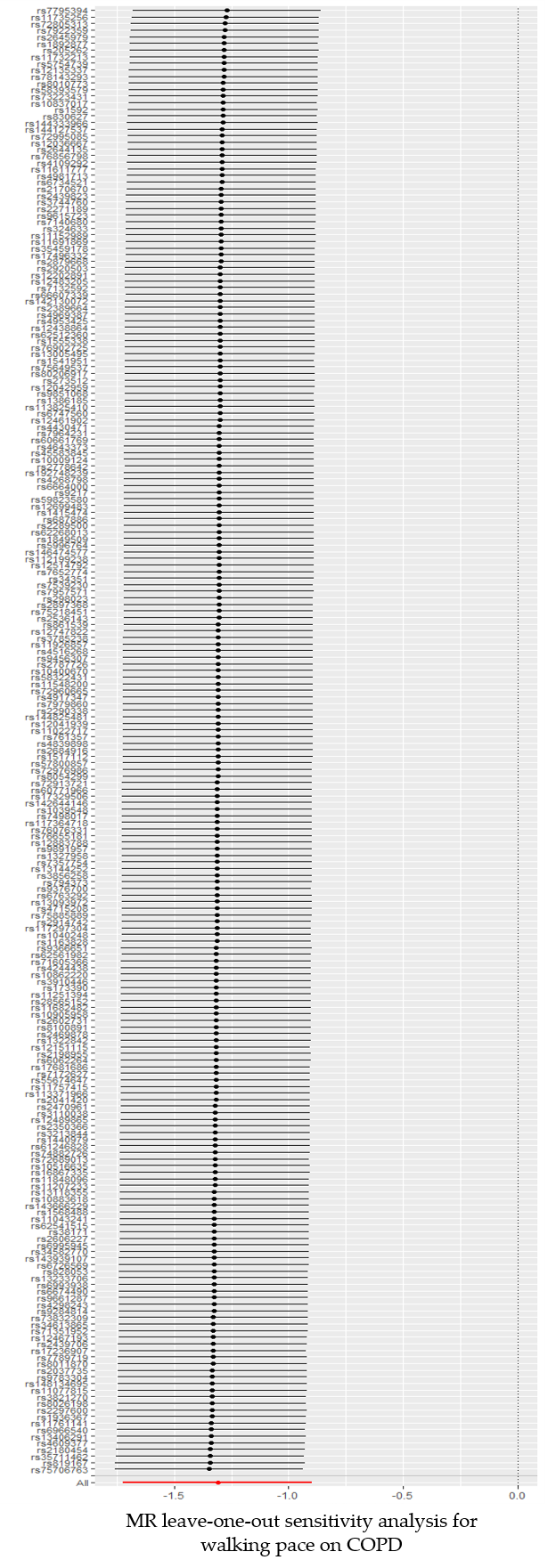


(A)


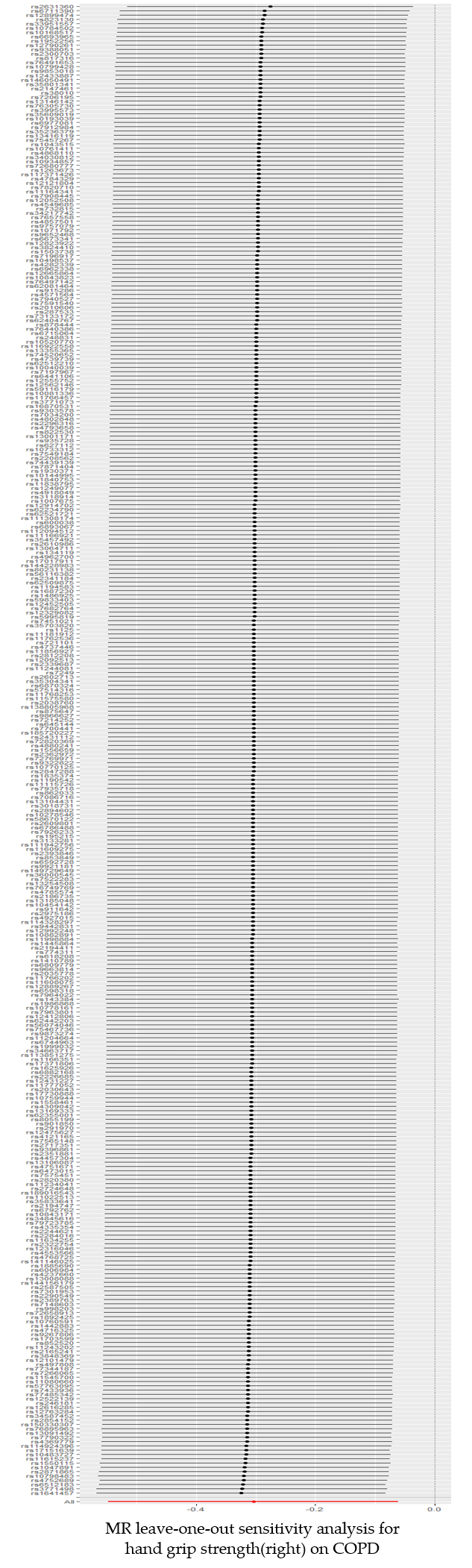


(B)


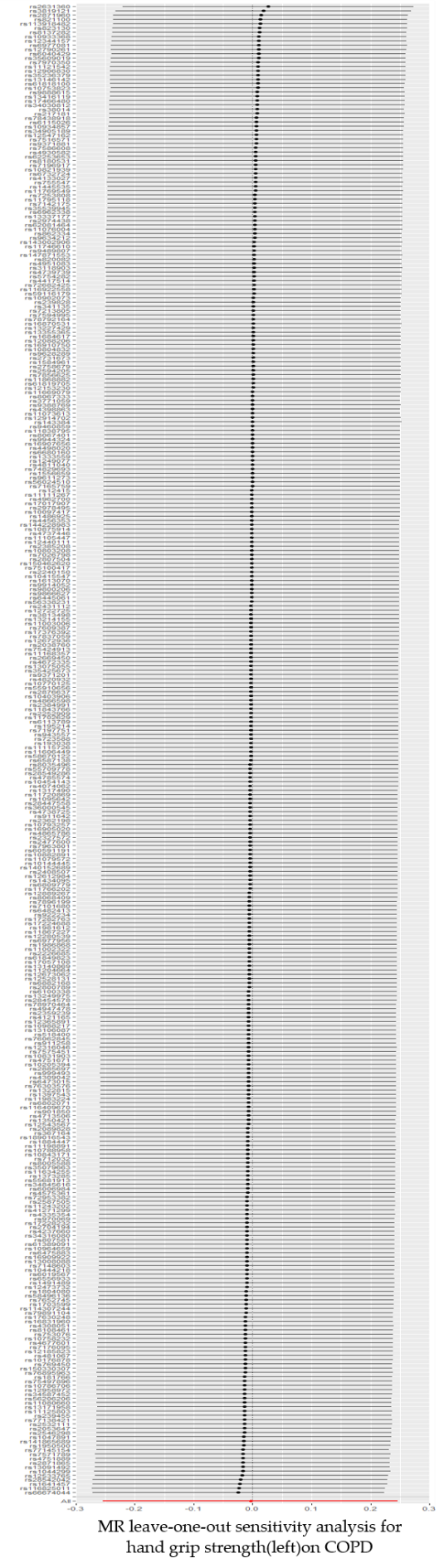


(C)

**Figure S3.** Leave-one-out sensitivity analysis of inverse MR analysis under the locus-wide significance threshold instrumental variables: (A) COPD on walking pace; (B) COPD on hand grip strength (right); (C) COPD on hand grip strength (left).


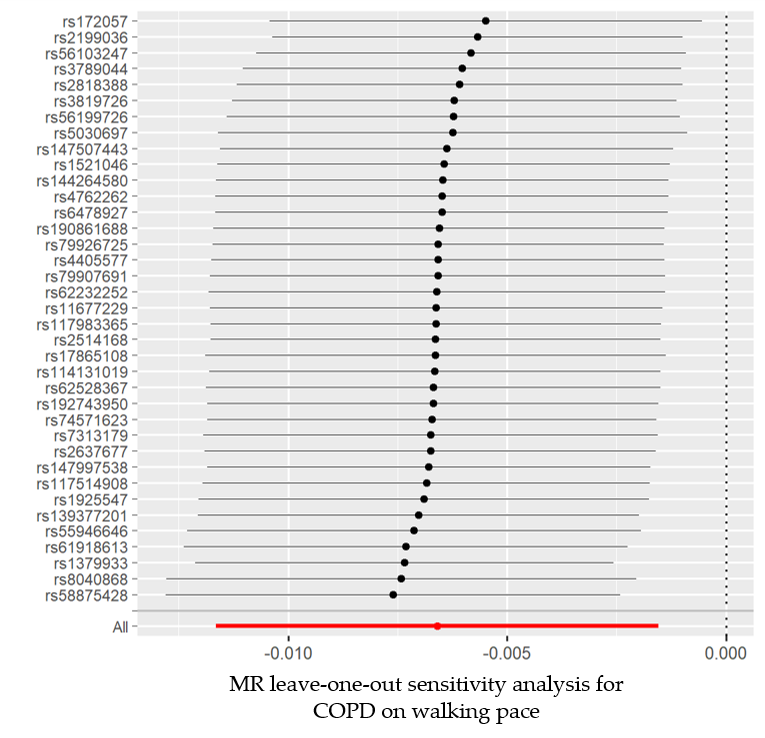


(A)


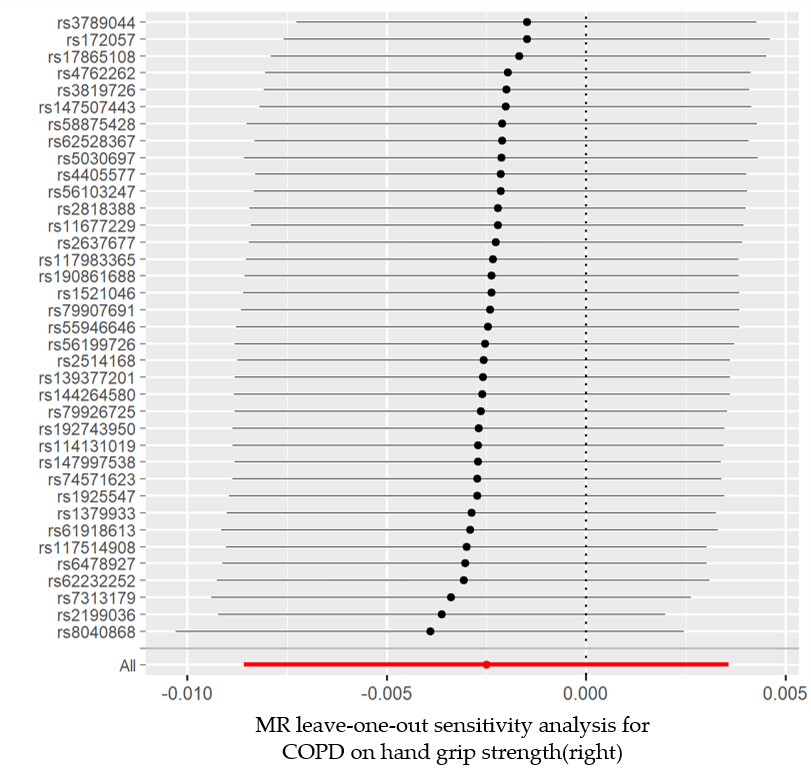


(B)


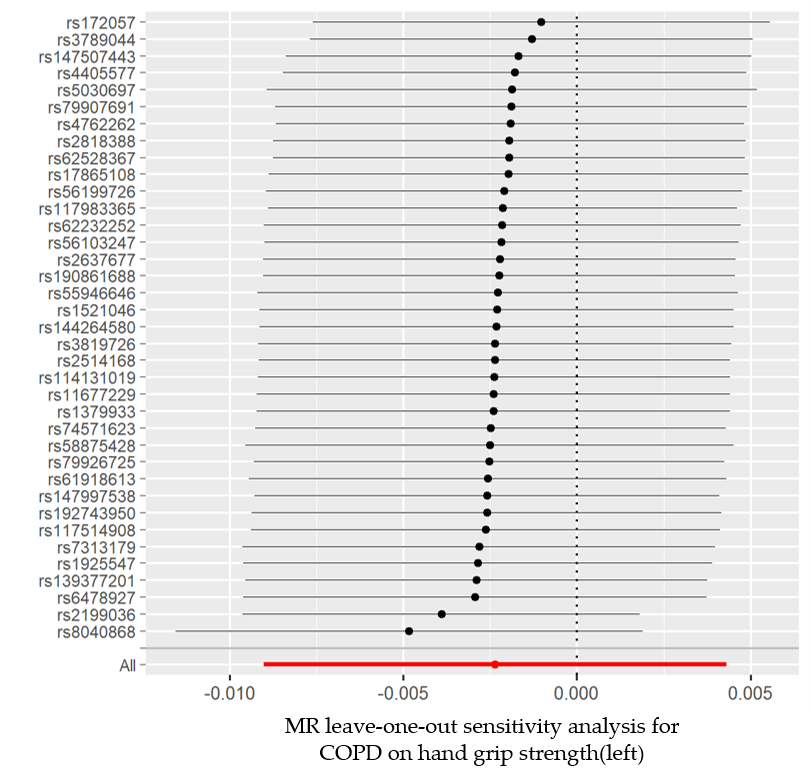


(C)
